# Students’ and lecturers’ perspective on the implementation of online learning in dental education due to SARS-CoV-2 (COVID-19): a cross-sectional study

**DOI:** 10.1186/s12909-020-02266-3

**Published:** 2020-10-09

**Authors:** Maximiliane Amelie Schlenz, Alexander Schmidt, Bernd Wöstmann, Nobert Krämer, Nelly Schulz-Weidner

**Affiliations:** 1 Department of Prosthodontics, Dental Clinic, Justus Liebig University, Schlangenzahl 14, 35392 Giessen, Germany; 2 Department of Pediatric Dentistry, Dental Clinic, Justus Liebig University, Schlangenzahl 14, 35392 Giessen, Germany

**Keywords:** Dentistry, Dental education, Online learning, Dental students, Dental lecturers, Questionnaires, COVID-19, Coronavirus

## Abstract

**Background:**

On account of physical distancing measures, universities worldwide are strongly affected by SARS-CoV-2 (COVID-19). Thus, the dental school of Justus-Liebig-University Giessen (Germany) transferred the established “face-to-face” learning to online learning in the spring term 2020. The aim of this study was to assess the students’ and lecturers’ perspectives on the implementation of online learning due to COVID-19, using a questionnaire survey.

**Methods:**

After the online period, all students and lecturers were asked to fill out an online questionnaire containing evaluative statements regarding *handling, didactic benefit*, *motivation,* and *overall assessment*. Furthermore, the questionnaire for lecturers contained additional aspects regarding *knowledge gain* in terms of providing online learning. Besides that, students and lecturers were asked for the amount of online learning in the future curriculum (independent of COVID-19). Data were subjected to regression analysis and T-test (*p* < .05).

**Results:**

36.8% of students preferred “face-to-face” learning instead of sole online learning. An increase of know how concerning online teaching was observable for lecturers. Both, students and lecturers, want to keep up with online courses in the future curriculum. However, in terms of the optimal amount of online learning a significant difference between students’ and lecturers’ perspective was observed. While students suggested 53.2% (24.9) (mean (standard deviation)) lecturers only stated 38.6% (21.5).

**Conclusions:**

Within the limitation of this study, students’ and lecturers’ showed a predominantly positive perspective on the implementation of online learning, providing the chance to use online learning even beyond COVID-19 in the future curriculum.

## Background

More than 95% of all countries worldwide reported infections with the severe acute respiratory syndrome coronavirus 2 (SARS-CoV-2) described as coronavirus disease 2019 (COVID-19) [1–3]. Therefore, most countries put physical distancing measures (e.g., closing of public, cultural, and educational institutions) in place to decelerate the infection rate [4–6]. Consequently, dental education at universities worldwide is strongly affected by the COVID-19 pandemic [7, 8] and dental schools have to face a new challenge to implement “distance learning” tools to continue dental education, which is strongly demanded by students [9, 10].

Earlier concepts during SARS-CoV-1 infection in 2003 [11, 12] or present recommendations by Wuhan University [13] propose online teaching as a mean of choice to prevent spreading of the virus by students. Even though some universities already implemented online courses before the COVID-19 pandemic [14], normally dental education in Germany features “face-to-face” teaching. But also in other countries, digitalization at dental schools has been characterized as a slow process [14]. However, this pandemic does not only create the need but rather may provide the chance to accelerate digital transformation in medical education [8, 15]. This could have a positive effect on future dental education even beyond COVID-19. Nevertheless, practical training on manikins in the preclinical curriculum and patient treatment in the clinical curriculum is indispensable for dental education. That means online learning is only applicable to theoretical learning content.

It is accepted that students’ assessments is the important factor of the benefits and value of online learning and the evaluation of their attitudes are important factors in assessing success [16]. Nevertheless, most studies solely focussed on students perspectives [17–20] and to the best of our knowledge, no survey regarding the implementation of online learning in dental education considered both students’ and lecturers’ perspectives [13]. However, for efficient further development of online courses both perspectives have to be considered.

Therefore, the aim of this study was to evaluate the students’ and lecturer’s perspective towards our new online learning courses through two online questionnaires (one for students and one for lecturers) in the challenging time of the SARS-CoV-2 pandemic.

## Methods

### Teaching concept of the Giessen dental school

Before the COVID-19 pandemic, practical preclinical and clinical courses were accompanied by theoretical “face-to-face” courses distributed over a 16-week semester. The teaching included both preclinical and clinical training on manikins. In the spring term 2020, the dental school of JLU decided to pool all theoretical courses over 4 weeks, in April and May 2020, to implement online learning instead of “face-to-face” courses. Thus, preclinical (1–5 semester) and clinical (6–10 semester) students stayed at home. To make lessons possible, synchronous formats such as live online courses were accomplished via a new online videoconference system (Webex Meetings, Cisco Systems, Dusseldorf, Germany). Whereas for asynchronous formats, such as dubbed lectures uploaded to online platforms for self-study, the established online platforms Knowledge-Based Medical Education (k-MED) and Stud.IP of JLU was used. Furthermore, a combination of synchronous and asynchronous formats (e.g. lectures and scripts on online platforms and “consultation hours” for students’ questions) were provided.

Before initiating online courses, we contacted the student council to ensure that all dental students had the technical requirements to access online learning from home. Furthermore, we started online learning with a technical introduction – so-called ‘tech-checks’ – in the first week, to introduce the new videoconference system to students and to check the sound and image quality.

### Online survey

Two online questionnaires (one for students and one for lecturers) were designed in cooperation with the Teaching Evaluation Service Center of the JLU and provided using an online survey tool (LimeSurvey, Hamburg, Germany) (Footnotes). The questionnaires contained evaluative statements regarding handling (the way students or lecturer deals with online learning), didactic benefit (the way students or lecturer indented online learning as helpful regarding dental education), and motivation (the way students or lecturer were enthusiasm for online learning). Furthermore, the questionnaire for lecturers contained additional aspects regarding the implementation and knowledge gain in terms of providing online learning. All study participants could agree or disagree with the statements using a five-point Likert scale [21] and were asked whether and to what extent “face-to-face” and online learning differed. For latter participants could decide whether they prefer online learning (1), equivalent (2) or “face-to-face” (3). Also, they were asked about the preferred amount of online learning in the future curriculum (independent of COVID-19). Finally, demographic questions were asked.

The questionnaires were evaluated anonymously, and abstention was allowed for each statement. The study was conducted in accordance with the ethical standards of the Institutional Review Board and the local ethics committee of the JLU (Ref. no. 84/20).

All dental students participating in the spring term 2020 (*n* = 299) and all lecturers (*n* = 47) with at least 1 year of teaching experience were included in this study. The online survey started after the end of the online learning period on May 27, 2020, and participants were recruited via e-mail. After 4 and 8 days, a reminder was sent to all potential participants. The survey was closed on June 5, 2020. Only fully completed questionnaires were included. Statistical analysis was performed using SPSS Statistics (version 24, IBM, Armonk, NY, USA). The distribution of responses was presented as mean and standard deviation. Data on the three groups handling, didactic benefit, and motivation of students’ perspectives were further statistically analyzed. Therefore, principle axis factoring with rotation oblimin was conducted. Considering the aspects of intrinsic value, scree plot and content, two factors were applied. Cronbach‘s Alpha showed acceptable reliability for pooled data of handling (.729) and didactic benefit (.659) (Table 1). Regression analysis was performed to analyze the association with gender and semester for the three groups. T-test was applied to analyze data regarding the amount of online learning in the future curriculum (*p* < .05).

**Table 1:** Factor loadings (pattern matrix)

	Factor	
	1	2
H3: The online learning was structured well.	0.709	0.012
H2: I was able to prepare myself well in advance for the online learning (by script or book).	0.608	0.103
H5: The image and sound quality of online learning was good.	0.578	−0.040
H1: The technical introduction (‘tech-checks’) in the first week of the semester prepared me well for online learning.	0.571	−0.053
H4: The aspiration level of online learning was good.	0.553	−0.106
D1: In the current situation, online learning was a good option for learning the theoretical part of education.	0.262	−0.594
D4: I generally prefer “face-to-face” rather than online learning.	−0.076	0.478
D5: I do not think that online learning is useful and would have preferred a ‘non-semester’ and (if possible) continuing with ‘normal’ “face-to-face” learning in winter semester.	−0.145	0.477
D2: By participating in the online learning, I feel well prepared for the practical part of education.	0.448	−0.464
D3: In the context of online learning I dare to ask questions more often than “face-to-face”.	−0.124	−0.412
Eigenvalues	3.65	1.35
% of variance	36.51	13.46
Cronbach’s alpha	0.729	0.659

## Results

A total of 242 (166 female, 69 male, 7 no answer) students completed the questionnaire, which represented a response rate of 80.9%. Concerning gender and semester distribution, there were no significant differences compared to the basic group (*n* = 299, chi-squared test, *p* > .05). Among them, 83.5% participated in all the online learning courses and 12.8% stated that they participated in the majority of courses.

In general, students assessed the aspects of handling, didactic benefit, and motivation mostly positive (Table 2). The majority of students felt well prepared by the tech-checks but had difficulties to prepare themselves sufficiently in advance for online learning. However, most students agreed that online learning was well-structured and the level of ambition was good, which means that they could follow the teaching content and did not feel over- or unchallenged. Furthermore, the image and sound quality were rated positively (Table 2).

**Table 2:** Items and descriptive statistics of students’ questionnaire

Item	Item description	M	SD	N
Handling^a^	H1: The technical introduction (‘tech-checks’) in the first week of the semester prepared me well for online learning.	4.28	0.97	224
	H2: I was able to prepare myself well in advance for the online learning (by script or book).	3.45	1.14	230
	H3: The online learning was structured well.	4.31	0.89	230
	H4: The aspiration level of online learning was good.	4.42	0.82	233
	H5: The image and sound quality of online learning was good.	4.17	0.78	234
Didactic benefit^a^	D1: In the current situation, online learning was a good option for learning the theoretical part of education.	4.69	0.7	236
Didactic benefit^a^	D2: By participating in the online learning, I feel well prepared for the practical part of education.	3.73	1.02	227
Didactic benefit^a^	D3: In the context of online learning I dare to ask questions more often than “face-to-face”.	2.89	1.26	224
Didactic benefit^a^	D4: I generally prefer “face-to-face” rather than online learning.	2.98	1.36	233
Didactic benefit^a^	D5: I do not think that online learning is useful and would have preferred a ‘non-semester’ and (if possible) continuing with ‘normal’ “face-to-face” learning in winter semester.	1.39	0.9	231
Motivation^a^	M1: The use of new digital teaching methods (e.g. online teaching) motivates me to learn.	3.78	1.2	235

*M* mean, *SD* standard deviation, *N* number of valid answers (total: *N* = 242)

^a^ type of answer: 1 = strongly disagree, 5 = strongly agree

Concerning students’ learning preferences, almost all students found that online learning was a good option in this special time of the COVID-19 pandemic. However, many students stated that they did not feel well prepared for the practical part of the curriculum by solely participating in online learning. A good note was that students interacted with lecturers by asking questions through the online platforms. Even though 36.8% of students preferred “face-to-face” courses instead of online teaching alone, only 5.6% stated that online learning was not useful. More than half of the students agreed that using online platforms motivates them to learn (Table 2).

There was a significant correlation between the aspects handling, didactic benefit, and motivation (Table 3). Furthermore, the regression analysis of handling, didactic benefit, and motivation of students’ perspectives revealed a significant association with semesters (*p* < .0001). However, an association with gender was only found for the didactic benefit. Female students agreed with the statements regarding the didactic benefit of online learning significantly more than male students (*p* = .010).

**Table 3:** Items and descriptive statistics of lecturers’ questionnaire

Item	Item description	M	SD	N
Didactic benefit^a^	D1: In the current situation, online learning was a good way to teach the theoretical part of education.	4.6	0.56	30
Didactic benefit^a^	D2: The theoretical teaching content could be covered just as well by online teaching formats as it would have been possible in a classroom course (lecture/seminar).	3.52	1.33	29
Didactic benefit^a^	D3: I found the students during the online teaching to be disciplined and attentive.	4.04	0.87	25
Didactic benefit^a^	D4: I feel more uncomfortable using new teaching methods such as online teaching than in “face-to-face” teaching such as lectures because I miss direct communication with the students.	3.03	1.4	29
Didactic benefit^a^	D5: I do not think that online learning is useful and would have preferred a ‘non-semester’ and (if possible) the continuation of ‘normal teaching’ in the winter semester.	1.81	1.2	31
Motivation^a^	M1: The use of new digital teaching methods (e.g. online teaching) motivates me.	3.93	1.1	30
Motivation^a^	M2: The online learning formats are an added value, because I can also use them for other training purposes (e.g. conventions).	3.25	1.48	28

*M* mean, *SD* standard deviation, *N* number of valid answers (total: *N* = 35)

^a^ type of answer: 1 = strongly disagree, 5 = strongly agree

Regarding the technical devices, the majority of students used laptop computers (69.8%) for online learning, followed by tablets (16.5%), smartphones (7%), and desktop computers (4.6%). Most students participated from home (57.0%) and used wireless local area networks (LAN) (87.6%) for connection. Over 95% of students stated that they did not have major problems with an internet connection.

A total of 35 (74.5%) lecturers (18 female, 16 male, 1 no answer) responded to the survey. Concerning gender, there was no significant difference compared to the basic group (*n* = 47, chi-squared test, *p* > .05). Among them, 6 professors, 4 Ph.D. candidates, 1 post-doc, 23 members of the teaching staff, and 1 Associated professor fully completed the survey with teaching experience between 1 and 40 years (median: 8 years, interquartile range: 7.08–14.01).

In total, 60.0% of lecturers had never dealt with online learning formats before the COVID-19 pandemic. Only 14.3% of lecturers had experience with online learning before (e.g. attending trainings or conventions, self-studied, listened to reports from colleges or already taught online). In times of COVID-19, most of the lecturers used synchronous online learning formats (54.3%), followed by a combination of synchronous and asynchronous formats (20.0%) and asynchronous formats alone (5.7%). Regarding the technical devices, laptop computers (51.4%) and desktop computers (34.3%) were mainly used. In addition to wireless LAN (28.6%), LAN (62.9%) was used for the majority of internet connections with mainly no interruptions in connection (62.9% never, 20.0% in a minority of cases). In 17.1% of cases, online teaching took place at home (home office), while 60.0% of the lecturers conducted online learning courses from their office at the dental clinic. Only 2.9% of them used specially equipped conference rooms in dental clinics.

The results regarding the aspects of didactic benefit and motivation are given in Table 3. In the time of COVID-19, online learning was useful as a substitute for “face-to-face” learning, as strongly agreed by 57.1% of lecturers and agreed by an additional 5.7% of them. The majority of lecturers quickly adapted to online learning. Furthermore, many lecturers stated that it was very straightforward to transfer their “face-to-face” teaching content to online formats. Moreover, 25.0% disagreed with the need for more support for more time to prepare online learning courses. Nearly 60.0% perceived the cooperative character during the implementation of online learning predominantly positive. Regarding the didactic benefit, 57.1% agreed with the implementation of online learning due to COVID-19 and saw a good way to teach the theoretical classes through online courses (54.3% strongly agreed, 28.6% agreed) without a loss of content. Although lecturers acknowledged the discipline of the majority of the students, they stated feeling more comfortable in “face-to-face” teaching. However, most lecturers were motivated by using online learning, and some even had added value by using the prepared online learning courses for training purposes.

Regarding knowledge gain before and after online teaching, a few lecturers were more favorable for online teaching afterward, where as the motivation for teaching was almost unchanged. However, an increase was observable regarding knowledge gain.

Table 4 demonstrate the overall assessment about the differences between online and “face-to-face” learning comparing students’ and lecturers’ perspective.

**Table 4:** Items and descriptive statistics of overall assessment (students and lecturers)

Students’ perspective			Overall assessment ^**a**^:Please assess whether and to what extent “face-to-face” and online learning differ regarding the following aspects: (1 = online learning, 2 = equivalent, 3 = “face-to-face”)	Lecturers’ perspective		
M	SD	N		M	SD	N
2.45	0.61	222	More modern	2.5	0.51	30
2.0	0.79	228	More fun	1.57	0.5	30
1.78	0.64	218	More tips of lecturers	1.38	0.61	32
1.78	0.7	229	Queries better possible	1.53	0.72	32
1.89	0.7	229	Better knowledge transfer	1.69	0.64	32
2.69	0.61	230	Easier participation	1.87	0.75	32
2.51	0.69	227	Less time effort	1.72	0.63	32

^a^ type of answer: 1 = online learning, 2 = equivalent, 3 = “face-to-face”

With regard to the aspects: more modern, tips and feedback, queries, and knowledge transfer, a high consensus between students and lecturers was noticed. Nevertheless, students stated that they had more fun, could better participate, and invested less time with online learning. Obviously, lecturers had a different opinion on those aspects, especially regarding easy participation and time effort (Table 4).

In terms of the optimal amount of online learning for further implementation of a curriculum, independent of COVID-19, a significant difference between students’ and lecturers’ perspective was observed (*p* = .002). Students demand 53.2% (mean) (standard deviation: 24.9) of the theoretical part in terms of online learning, while lecturers only want 38.6% mean (standard deviation: 21.5) of the theoretical curriculum online.

## Discussion

During the COVID-19 pandemic, physical distancing measures are required and have an enormous impact on dental education [7, 8]. Because online learning allows participating in learning activities independent of the location [22], the JLU implemented online learning in the spring semester 2020 to prevent the spread of the virus. Thus, most students stayed at home and did not return from the spring break.

Before initiating online courses, we ensured that all students had unrestricted access to online learning and questionnaire survey. Furthermore, the high response rate reduces the bias of not representative data. Concerning gender distribution and semester, there were no significant differences observed when compared to the basic group (chi-squared test, *p* > .05). All participants (students and lecturers) were informed that data collection was completely anonymous and did not allow any traceability of the answering person. Thus, the bias in answering was prevented. However, a clear limitation of this cross-sectional study is that students and lecturers of only one dental school at one point were asked to participate in this study. Therefore, other universities should also be surveyed in future studies.

To achieve the highest possible educational success, it is important to involve all participants in the teaching process [16, 23]. Therefore, the implementation of online learning due to COVID-19 was evaluated using a Likert scale, the standard procedure for surveys in the field of medicine [17, 19, 24, 25].

As described by Asiry [25], ease of access is important for online learning. The university ensured that every dental student had access to online learning before starting online courses. Therefore, it was helpful that the videoconference tool Webex Meeting was app-based, so even students without a desktop or laptop computer could participate in synchronous online learning using a tablet or smartphone, using a wireless LAN connection. In addition, the tech-checks were helpful and evaluated mainly positive by students. Therefore, it can be useful to implement a technical check before starting with online learning.

Many students stated that participation in online learning was easier compared to “face-to-face” courses. This might explain the high participation in online learning among all semesters.

Even though most students agreed that online learning at times of COVID-19 pandemic was useful and they would not prefer a ‘non-semester’, many students did not feel well prepared for practical courses with online learning alone. These findings are in good accordance with the literature [9, 10]. Besides theoretical education, dentistry requires manual training and clinical patient care; therefore, “face-to-face” teaching is important. However, the positive-rated aspects of online learning, such as a higher motivation of students, easier participation, and less time effort can be used to improve the future dental curriculum.

Although the required transfer from “face-to-face” teaching to online learning accelerated the digitalization process in dental education [8, 15], the complete changeover showed that students and lecturers had difficulties in preparing in advance for online learning.

Although over 60% of lecturers at our dental school had no experience with online teaching before the COVID-19 pandemic, lecturers adapted very quickly to online learning, and the knowledge gain regarding the implementation of online learning was very high.

Similar to the students, most lecturers also found that online learning was a good alternative during the COVID-19 pandemic. However, lecturers had a different opinion about the positive-rated aspects (less time effort, easier participation, and more fun) by students regarding online learning. Obviously, the transfer from “face-to-face” to online learning was time-consuming and, because of various functions in dental clinics, most lecturers cannot work from home.

Independent of COVID-19, students indicated a mean amount of 53.2% (standard deviation: 24.9) regarding the theoretical part in terms of online learning for the future curriculum, and lecturers indicated only 38.6% (21.5). Even though a significant difference between students’ and lecturers’ regarding the amount of online learning in the future curriculum was observed, both groups strongly suggested a continuation of online learning. Furthermore, students in higher semesters rated online learning regarding the aspects handling, didactic benefit, and motivation higher than students in lower semesters. These are important facts that should be considered in the future. Currently, synchronous online courses are used mostly, but in the future a combination of online and attendance (“face-to-face”) learning, such as problem-based learning or flipped classroom methods, can be implemented more in dental education [9, 26]. However, as indicated by lecturers, this requires personnel and financial support [14, 26].

Subsequent to the online learning period, a step-wise concept back to “face-to-face” teaching started to enable also practical education during COVID-19 pandemic [12]. Therefore, students began to exercise in small groups on manikins and will start to practice on patients in July 2020. This concept is in accordance with the way HongKong University reacted during the SARS-CoV-1 outbreak in 2003 [11, 12]. However, compared to past outbreaks, today digital technology currently allows for an almost real-time record of infections and a sharing of information around the globe [3].

There is probably no gold standard or one way to deal with this pandemic situation in the education of dental students. Even though the increase in COVID-19 cases has slowed down, the authors hypothesize that it will take time to return to ‘normal teaching’. Therefore, we better develop new and more flexible concepts/curricula with a high amount of online learning, without “face-to-face” contact. The results of our survey at an early stage should highlight the steps taken to manage the crisis and point out the opportunity to minimize the impact of such outbreaks on teaching in dentistry and be better prepared for similar disruptions in the future.

## Conclusions

Within the limitation of this study, students’ and lecturers’ showed a predominantly positive perspective on the implementation of online learning, providing the chance to use online learning even beyond COVID-19 in the future curriculum.

## Supplementary information

**Additional file 1.** Questionnaire Students.

## Data Availability

The datasets of this article are available from the corresponding author on a reasonable request.

## Abbreviations

SARS-CoV-2: severe acute respiratory syndrome coronavirus 2
COVID-19: coronavirus disease 2019
k-MED: Knowledge-based Medical Education
