# Supplementary material for: Students’ and lecturers’ perspective on the implementation of online learning in dental education due to SARS-CoV-2 (COVID-19): a cross-sectional study
Source: BMC Med Educ. 2020 Oct 9;20:354. doi: 10.1186/s12909-020-02266-3 (PMC7545382; doi:10.1186/s12909-020-02266-3)
Supplement: Supplementary file 1 — Additional file 1. Questionnaire Students. [file 12909_2020_2266_MOESM1_ESM.docx]

Questionnaire Students

Handling

1. How often did you participate on the online learning courses?

Please select one of the following answers:

- not at all
- in minority
- nearly half
- predominantly
- full
- no answer

2. To what extent do the following statements apply to the current online learning?

Please select the appropriate answer for each point:

|  | strongly disagree | disagree | neutral | agree | strongly agree | no answer |
| --- | --- | --- | --- | --- | --- | --- |
| The technical introduction (‘tech-checks’) in the first week of the semester prepared me well for online learning. | 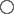 | 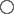 | 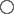 | 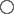 | 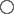 | 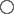 |
| I was able to prepare myself well in advance for the online learning (by script or book). | 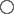 | 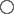 | 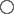 | 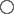 | 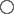 | 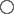 |
| The online learning was structured well. | 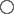 | 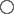 | 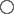 | 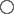 | 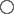 | 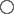 |
| The aspiration level of online learning was good. | 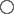 | 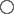 | 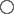 | 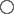 | 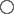 | 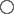 |
| The image and sound quality of online learning was good. | 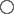 | 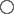 | 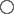 | 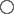 | 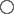 | 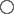 |

Didactic benefit and motivation

3. To what extent do the following statements apply to the current online learning?

Please select the appropriate answer for each point:

|  | strongly disagree | disagree | neutral | agree | strongly agree | no answer |
| --- | --- | --- | --- | --- | --- | --- |
|  | 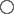 | 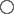 | 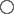 | 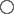 | 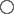 | 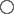 |
| In the current situation, online learning was a good option for learning the theoretical part of education. | 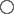 | 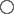 | 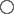 | 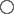 | 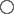 | 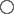 |
| By participating on the online learning, I feel well prepared for the practical part of education. | 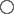 | 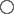 | 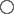 | 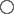 | 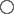 | 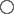 |
| The use of new digital teaching methods (e.g. online teaching) motivates me to learn. | 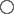 | 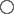 | 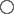 | 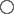 | 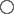 | 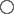 |
| In the context of online learning I dare to ask questions more often than face-to-face. | 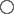 | 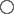 | 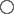 | 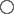 | 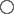 | 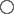 |
| I generally prefer face-to-face rather than online learning. | 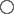 | 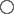 | 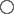 | 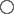 | 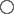 | 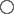 |
| I do not think that online learning is useful and would have preferred a ‘non-semester’ and (if possible) continuing with ‘normal’ face-to-face learning in winter semester. | 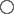 | 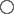 | 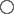 | 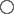 | 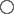 | 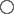 |

Overall assessment

4. Please assess whether and to what extent face-to-face and online learning differ regarding the following aspects:

Please select the appropriate answer for each point:

|  | face-to-face | equivalent | online learning | No answer |
| --- | --- | --- | --- | --- |
| Less time effort | 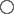 | 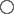 | 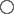 | 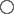 |
| easier participation | 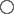 | 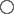 | 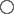 | 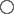 |
| better knowledge transfer | 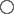 | 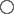 | 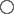 | 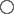 |
| questions better possible | 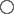 | 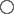 | 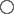 | 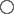 |
| More tips of lecturers | 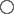 | 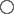 | 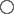 | 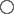 |
| more fun | 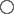 | 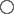 | 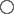 | 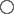 |
| more modern | 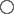 | 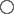 | 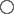 | 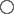 |

5. How large should be the amount of online learning regarding the theoretical part of education (independent of COVID-19) in the future curriculum?

Please enter your answer here:

____ Percent (%)

Numbers in percent from 0 to 100

Final questions

6. What is your sex?

Please select only one of the following answers:

- male
- female
- inter / diverse
- no answer

7. In which semester are you currently studying?

Please enter your answer here:

____ semester

8. Which device did you mainly use for the participation on the online learning courses?

Please select only one of the following answers:

- Smartphone
- Tablet
- Laptop
- PC
- No answer

9. What type of internet connection did you use mostly?

Please select only one of the following answers:

- Mobile network
- W-LAN
- LAN
- No answer

10. How often did you have problems with your internet connection so that you could not follow relevant content properly?

Please select only one of the following answers:

- never
- among the minority of events
- about half of the events
- for the majority of events
- always
- no answer

11. Where did you spend most of your time during online learning?

Please select only one of the following answers:

- Giessen
- Home town
- No answer

Questionnaire Lecturers

Handling

1. To what extent do the following statements apply to you?

Please select one or more items from the list.

I have been actively involved in online teaching formats for the past 2 years by...

- …attending trainings at the university (e.g. HDM, ZfbK, Lehre 4.0).
- …attending conventions on teaching development or similar.
- …self-studying (books, internet, publications, etc.).
- …listening to reports from colleagues, but had no time to implement my own ideas to my courses.
- …already teaching completely or partially with online learning.
- No statement is correct, because I never dealt with online teaching formats before Covid-19.
- No answer.

2. What format of online learning did you use?

Please choose one of the following answers:

- Synchronous formats like live online seminars/lectures (e.g. Webex).
- Asynchronous formats like dubbed lectures uploaded to online platforms for self-study (e.g. k-MED, Stud-IP).
- Combination of synchronous and asynchronous formats (e.g. lectures and scripts on online platforms and "consultation hours" for students' questions).
- No answer.

3. To what extent do the following statements apply to the current online learning?

Please select the appropriate answer for each point:

|  | strongly disagree | disagree | neutral | agree | strongly agree | No answer |
| --- | --- | --- | --- | --- | --- | --- |
| I adapted very quickly to online learning. |  |  |  |  |  |  |
| It was very straightforward to transfer my teaching content to online formats. |  |  |  |  |  |  |
| I would have liked more support from the university in implementing my online learning formats. |  |  |  |  |  |  |
| The preparation of online courses was more time-consuming than for dace-to-face courses. |  |  |  |  |  |  |

Didactic benefit and motivation

4. To what extent do the following statements apply to the current online learning?

Please select the appropriate answer for each point:

|  | strongly disagree | disagree | neutral | agree | strongly agree | No answer |
| --- | --- | --- | --- | --- | --- | --- |
| In the current situation, online learning was a good way to teach the theoretical part of education. |  |  |  |  |  |  |
| The theoretical teaching content could be covered just as well by online teaching formats as it would have been possible in a classroom course (lecture/seminar). |  |  |  |  |  |  |
| I found the students during the online teaching to be disciplined and attentive. |  |  |  |  |  |  |
| The use of new digital teaching methods (e.g. online teaching) motivates me. |  |  |  |  |  |  |
| I feel more uncomfortable using new teaching methods such as online teaching than in face-to-face teaching such as lectures because I lack direct communication with the students. |  |  |  |  |  |  |
| I do not think that online learning is useful and would have preferred a ‘non-semester’ and (if possible) the continuation of ‘normal teaching’ in the winter semester. |  |  |  |  |  |  |

Overall assessment

5. Please assess whether and to what extent face-to-face and online learning differ regarding the following aspects:

Please select the appropriate answer for each point:

|  | face-to-face | equivalent | online learning | No answer |
| --- | --- | --- | --- | --- |
| Less time effort |  |  |  |  |
| easier participation |  |  |  |  |
| better knowledge transfer |  |  |  |  |
| questions better possible |  |  |  |  |
| More tips for students |  |  |  |  |
| more fun |  |  |  |  |
| more modern |  |  |  |  |

6. How large should be the amount of online learning regarding the theoretical part of education (independent of COVID-19) in the future curriculum?

Please enter your answer here:

____ Percent (%)

Numbers in percent from 0 to 100

Knowledge gain

7. To what extent do the following statements about online teaching apply to you?

Please place 2 crosses per line.

Please select the appropriate answer for each point:

|  | BEFORE online teaching… | | | | |  | AFTER online teaching… | | | | |  |
| --- | --- | --- | --- | --- | --- | --- | --- | --- | --- | --- | --- | --- |
|  | strongly disagree | disagree | neutral | agree | strongly agree | No answer | strongly disagree | disagree | neutral | agree | strongly agree | No answer |
| I knew / know a lot about online teaching learning. |  |  |  |  |  |  |  |  |  |  |  |  |
| I could/can implement online learning courses. |  |  |  |  |  |  |  |  |  |  |  |  |
| I was/ am favourable to online teaching. |  |  |  |  |  |  |  |  |  |  |  |  |
| I was /am motivate for teaching. |  |  |  |  |  |  |  |  |  |  |  |  |

Final questions

8. What is your sex?

Please select only one of the following answers:

- male
- female
- inter / diverse
- no answer

9. I am currently...

Please select only one of the following answers:

- professor
- PHD candidate
- Post-doc
- Teaching staff
- Associated professor
- Project staff
- No answer

10. How many years of teaching experience do you have?

Please enter your answer here:

____ years

11. Which device did you use mainly for the implementation of the online teaching?

Please select only one of the following answers:

- Smartphone
- Tablet
- Laptop
- PC
- No answer

12. What type of Internet connection did you use most?

Please select only one of the following answers:

- Mobile network
- W-LAN (wireless connection)
- LAN (cable connection)
- No answer

13. How often did you have problems with your internet connection so that you could not follow relevant content properly?

Please select only one of the following answers:

- never
- among the minority of events
- about half of the events
- for the majority of events
- always
- no answer

14. Where did you spend most of your time during the online teaching?

Please select only one of the following answers:

- Home Office
- Own office in the university/ clinical centre
- Common rooms (lecture halls, conference rooms or similar)
- No answer
